# Occupational Ionizing Radiation-Induced Skin Injury Among Orthopedic Surgeons: A 5-Year Longitudinal Cohort Study

**DOI:** 10.2106/JBJS.OA.25.00332

**Published:** 2026-01-16

**Authors:** Gentaro Kumagai, Daiki Rokunohe, Eiji Sasaki, Chihiro Sagara, Toru Asari, Takahide Kaneko, Eijiro Akasaka, Yasuyuki Ishibashi

**Affiliations:** 1 Department of Orthopedic Surgery, Hirosaki University Graduate School of Medicine, Hirosaki, Aomori, Japan; 2 Department of Dermatology, Hirosaki University Graduate School of Medicine, Hirosaki, Aomori, Japan; 3 Department of Dermatology, Juntendo University. Urayasu Hospital, Urayasu, Chiba, Japan

## Abstract

**Background:**

This 5-year longitudinal study evaluated changes in occupational radiation exposure and radiation-induced skin injury among orthopaedic surgeons, focusing on the effects of educational campaigns.

**Methods:**

Orthopaedic surgeons at Hirosaki University were surveyed in 2019 and 2024. Self-reported weekly fluoroscopy (“beam-on”) time and dermatologist-graded hand skin findings were compared. Educational campaigns (2020-2023) emphasized As Low As Reasonably Achievable principles and personal protective equipment. We hypothesized that repeated education would improve radiation-safety attitude, reduce self-reported fluoroscopy time, and mitigate dermatologic injury.

**Results:**

The proportion of surgeons cautious about radiation increased from 5.8% to 70.9%. The median weekly self-reported fluoroscopy time decreased from 9.5 to 8.0 minutes (p = 0.045). The prevalence of radiation-induced skin injury declined from 34.9% to 25.6%. Inter-rater reliability was excellent (weighted κ = 0.910). Nonspine surgeon status predicted improvement in skin condition.

**Conclusions:**

During the 5-year period in which repeated radiation-safety education was conducted, surgeons demonstrated improved safety attitudes, decreased self-reported fluoroscopy time, and improved dermatologist-graded skin findings. These observations indicate an association between educational activities, self-reported exposure, and skin findings, but do not establish causality because exposure was self-reported and the study lacked a control group.

**Level of Evidence:**

Therapeutic Level III. See Instructions for Authors for a complete description of levels of evidence.

## Introduction

Ionizing radiation is indispensable in orthopedic surgery, particularly in spine and trauma procedures requiring fluoroscopy and x-ray imaging^1-3^. However, cumulative exposure represents a serious occupational hazard for surgeons, leading to chronic dermatitis and, in severe cases, malignant transformation^4,5^. Previous studies reported a high prevalence of radiation-related skin and ocular disorders^2-4^, emphasizing the need for preventive strategies. Despite these concerns, most studies were cross-sectional and did not assess long-term effects of education.

To address this gap, our group previously conducted a clinical survey that identified radiation-induced skin changes among orthopaedic surgeons and underscored the necessity of continuous education on radiation safety^4^. Since that initial survey, repeated educational campaigns emphasizing the “As Low As Reasonably Achievable” (ALARA) principles and appropriate use of personal protective equipment (PPE) have been implemented.

This 5-year longitudinal follow-up study aimed to assess changes in radiation-safety awareness, weekly radiation exposure time, and dermatologist-confirmed radiation-induced skin injury among orthopaedic surgeons.

We hypothesized that education-focused campaigns would be associated with (1) increased radiation-safety awareness, (2) decreased weekly fluoroscopy time, and (3) improved or stable dermatologist-graded hand skin findings over 5 years.

## Materials and Methods

### Study Design and Participants

**Fig. 1:**
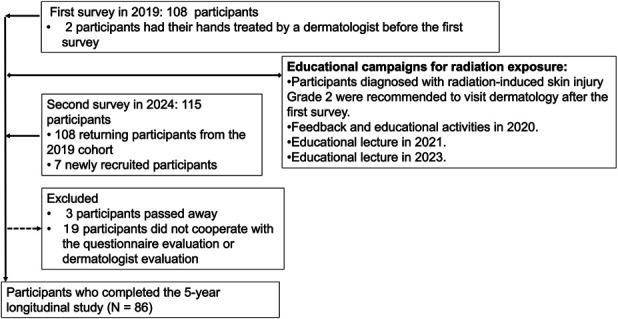
Flow diagram of the longitudinal study (showing participant inclusion/exclusion in 2019 and 2024 and educational campaigns for radiation exposure).

This 5-year longitudinal cohort study followed orthopedic surgeons at Hirosaki University who participated in the baseline 2019 survey (Asari et al.^4^). The second survey was conducted in 2024 to assess temporal changes in radiation-safety awareness, radiation exposure time, and dermatologist-confirmed skin injury. Of the 108 baseline participants, 86 surgeons completed both surveys and were included in the longitudinal analysis (follow-up rate, 79.6%). Educational campaigns were conducted between surveys (2020-2023) (Fig. 1). The study was approved by the University Ethics Committee (Approval No. 2024-009), and written informed consent was obtained at both time points. All data were anonymized in accordance with the Declaration of Helsinki^6^ and STROBE^7^.

### Educational Campaigns

Educational sessions (2020, 2021, and 2023) consisted of 30- to 60 minute-lectures with slides and case images covering ALARA principles, optimal positioning relative to the x-ray tube and detector, pulse/low-dose fluoroscopy settings, and appropriate use of PPE such as lead glasses, thyroid shields, and gloves. Each session emphasized risks to the eyes, thyroid, and hands. Attendance was mandatory (syllabus in Supplementary Appendix A).

### Questionnaire: Awareness and Exposure

Surgeons completed identical self-administered questionnaires in 2019 and 2024.

To assess awareness, participants were asked:

“In your daily practice, how careful are you about occupational radiation exposure?”

Responses were graded as *Never, A little, Neither, Sometimes careful,* or *Always careful.*

Weekly radiation exposure was self-reported using the question:

“How many minutes per week are you actually exposed to ionizing radiation?”

Participants reported average cumulative fluoroscopy “beam-on” time per week during the preceding 3 months, excluding ultrasound or nonionizing modalities. Because this variable was self-reported and not dosimeter-based, potential recall bias was acknowledged, and the variable was treated as secondary (the complete questionnaire is available in Supplementary Appendix B).

#### Dermatologic Assessment

Standardized photographs of both hands (dorsal and lateral views) were obtained and independently graded by 2 board-certified dermatologists, with adjudication by a third when necessary.

Skin findings were classified using predefined clinical criteria^4^:

Grade 0 = no symptoms; Grade 1 = mild changes (pigmentation, hyperkeratosis, telangiectasia); and Grade 2 = severe chronic dermatitis or precancerous/cancerous lesions.

Inter-rater reliability was excellent (weighted κ = 0.910; 95% CI, 0.830-0.989).

Participants with Grade 2 or suspicious lesions were referred for dermatologic evaluation, and all consultations were performed by the same dermatologists.

When mild changes were observed, individualized feedback on radiation protection was provided (representative grading images and full diagnostic criteria are provided in Supplementary Appendix C).

Participants were also asked which hand they primarily used for fluoroscopy or C-arm manipulation (“which hand do you mainly use to handle instruments or the C-arm during radiation-related tasks?”). Skin findings were analyzed by laterality (right, left, both, or none). These laterality data were collected as part of the original 2019 and 2024 surveys but had not been reported previously; no additional data collection was performed for this revision.

### Statistical Analysis

The primary analysis compared paired data (2019 vs. 2024) among the 86 surgeons evaluated at both time points. Changes in categorical variables were assessed using Wilcoxon signed-rank tests as appropriate. Cross-sectional data from all participants (n = 108 in 2019; n = 115 in 2024) were summarized descriptively. Exact 95% CIs were reported for proportions, and logistic regression was used to identify predictors of improvement in skin injury (sex, years of experience, spine specialization, and exposure time). A p value < 0.05 was considered significant. Analyses were performed using SPSS v25 (IBM, Armonk, NY) (detailed formulas and additional sensitivity analyses are provided in Supplementary Appendix D).

## Results

### Sociodemographic Characteristics of Surgeon Participants

A total of 108 orthopaedic surgeons participated in the 2019 baseline survey and 115 in the 2024 follow-up. Of the 115 surgeons in 2024, 108 were returning participants from the 2019 cohort and 7 were newly recruited surgeons. Among the 108 baseline participants, 22 did not participate in the follow-up (3 deceased, 19 nonresponders), leaving 86 surgeons who completed both surveys, constituting the longitudinal cohort for within-surgeon comparisons (follow-up rate, 79.6%). Unless stated otherwise, all temporal comparisons were based on this cohort (n = 86). Descriptive data from all participants (n = 108 in 2019; n = 115 in 2024) were used for background reference. Table I presents the sociodemographic characteristics of the orthopedic surgeon participants in 2019 and 2024. This study included 8.1% women. The average number of years of experience among participants was 18.4 ± 11.7 years in 2019. The proportion of board-certified specialists increased from 68.6% in 2019 to 95.3% in 2024. This increase primarily reflects that several younger surgeons within the longitudinal cohort (n = 86) obtained board certification during the 5-year interval, rather than the inclusion of new participants. The most common subspecialty in this study was spine surgery. In 2019, 2 participants had a prior dermatologic consultation; 3 and 1 additional surgeons visited dermatology after the first and second surveys, respectively.

**TABLE I:** Sociodemographic Characteristics of Orthopaedic Surgeons (Longitudinal Cohort, n = 86): 2019 vs. 2024

Variables	2019	2024
Female, n (%)	7 (8.1)	7 (8.1)
Years in practice, mean ± SD	18.4 ± 11.7	23.2 ± 11.8
Specialization held, n (%)	59 (68.6)	82 (95.3)
Subspecialty		
Spine surgeon, n (%)	23 (39.0)	28 (34.1)
Joint surgeon, n (%)	10 (16.9)	10 (12.1)
Hand surgeon, n (%)	10 (16.9)	11 (13.4)
Sports surgeon, n (%)	9 (15.3)	17 (20.7)
RA surgeon, n (%)	4 (6.8)	3 (3.7)
Tumor surgeon, n (%)	2 (3.4)	4 (4.9)
Pediatric surgeon, n (%)	2 (3.4)	1 (1.2)
Trauma surgeon, n (%)	0 (0)	2 (2.4)
Others, n (%)	1 (1.7)	7 (8.5)
History of dermatologic treatment for the hands	2 (2.3)	-
Dermatology visits after the survey	3 (3.5)	1 (1.2

RA = rheumatoid arthritis.

Notes: Data are n (%) unless otherwise indicated. Counts and percentages reflect the same 86 individuals assessed at baseline (2019) and follow-up (2024).

#### Changes in Attitudes Toward Radiation Exposure

**Fig. 2:**
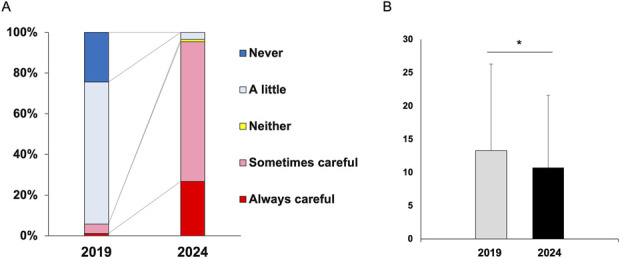
A. Changes in attitudes toward radiation exposure over the study period. Question stem: “In your daily practice, how careful are you about occupational radiation exposure?” Response options: Never, A little, Neither, Sometimes careful, Always careful. For paired binary analyses, responses were dichotomized as “Sometimes/Always careful” versus other categories. B. Radiation exposure time (min/week) comparison between 2019 and 2024.

The percentage of participants who reported being cautious about radiation exposure increased markedly from 5.8% in 2019 to 70.9% in 2024 (Fig. 2-A). This reflects improved awareness following the repeated educational interventions conducted between the 2 surveys.

### Weekly Radiation Exposure Time

The mean weekly radiation exposure time significantly decreased from 13.3 ± 13.0 min (95% CI, 10.6-16.1) in 2019 to 10.7 ± 10.9 min (95% CI, 8.4-13.0) in 2024 (p = 0.045, Wilcoxon signed-rank test; Fig. 2-B). The median values were 10.0 min (IQR 4-18) and 8.0 min (IQR 3-14), respectively.

### Changes in Dermatologist-Confirmed Radiation-Induced Skin Injury

**Fig. 3:**
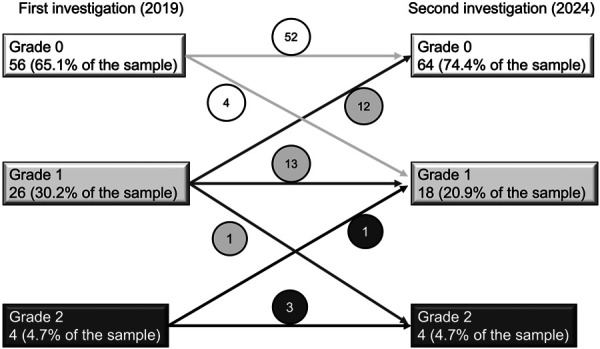
Changes in grades of radiation-induced skin injury diagnosed by dermatologists. All comparisons in Fig. 3 represent the paired cohort (n = 86).

Inter-rater agreement in 2024 was excellent (weighted κ = 0.910; 95% CI, 0.830-0.989), confirming consistency between dermatologists. The prevalence of radiation-induced skin injury decreased from 34.9% in 2019 to 25.6% in 2024 (Fig. 3). Five participants (5.8%) worsened, 68 (79.1%) were unchanged, and 13 (15.1%) improved. Among those initially at Grade 1, 12 improved to Grade 0, 13 remained Grade 1, and 1 progressed to Grade 2; no new Grade 2 lesions developed from Grade 0. Dermatology consultation among Grade 2 cases increased from 50% to 75%.

Radiation-induced skin injury by experience is summarized in Table II. Surgeons with ≤9 years had only Grade 0 to 1 lesions, and none progressed to Grade 2. Mild injuries declined in the 10–19-year group, while rates among those with ≥20 years remained stable, with Grade 2 lesions limited to this group. Although more advanced lesions were observed among surgeons with ≥20 years of experience, this pattern may reflect multiple factors—including age-related or non–radiation-related skin changes—and causality cannot be inferred from these data.

**TABLE II:** Changes in Radiation-Induced Skin Injury by Years of Experience (Longitudinal Cohort, n = 86)

	Grades of Radiation-Induced Skin Injury		
Years in Practice in 2019 (y)		2019	2024
≤9	Grade 0	24	25
	Grade 1	1	0
	Grade 2	0	0
			
10–19	Grade 0	14	17
	Grade 1	7	4
	Grade 2	0	0
			
20–29	Grade 0	10	13
	Grade 1	9	6
	Grade 2	1	1
			
30–39	Grade 0	7	8
	Grade 1	7	6
	Grade 2	3	3
			
≥40	Grade 0	1	2
	Grade 1	2	1
	Grade 2	0	0

Notes: Grades were assigned by dermatologists. Values show the number of the same individuals reclassified in 2024. Grade 0 = no clinical symptoms; Grade 1 = mild changes warranting observation; Grade 2 = severe chronic radiodermatitis or precancerous/cancerous lesions.

Injury occurred mainly on the dominant hand. Among 52 both-hand users, 13.5% showed injury; among right hand–dominant surgeons, 22.6% had right hand–only and 16.1% bilateral lesions, with none in left hand–dominant surgeons. Radiation-related skin findings were more common among surgeons with ≥20 years of experience, whereas no Grade 2 lesions occurred in those with ≤9 years of practice.

### Factors Related to Improvement in Radiation-Skin Injury

Univariate and multivariate logistic regression analyses demonstrated that not being a spine surgeon was significantly associated with improvement of radiation skin injury (odds ratio [OR] 0.236; 95% CI, 0.069-0.807; p = 0.021; multivariate OR 0.233; 95% CI, 0.062-0.878; p = 0.031; Table 3, see Supplementary Appendix). No other variables were significantly associated with improvement.

### Representative Cases

Representative cases are shown in Supplementary Appendix E (Fig. S1–S2). Hyperkeratotic lesions on the right index finger were excised surgically (Fig. S1), whereas longitudinal melanonychia of the right thumb regressed during the 5-year period. Another case demonstrated marked clinical improvement in mild hyperkeratosis, with almost complete resolution by 2024 (Fig. S2).

## Discussion

### Impact of Radiation Exposure on Skin Injury in Orthopedic Surgeons

This 5-year longitudinal study observed that, during a period in which repeated radiation-safety education was conducted, surgeons showed improved awareness and decreased self-reported fluoroscopy time. Dermatologist-graded skin findings also improved in many surgeons, although a subset—particularly spine surgeons—continued to show persistent lesions. This study demonstrates an association between self-reported fluoroscopy time and dermatologist-graded skin findings but does not establish a causal relationship. These findings indicate that increased awareness was associated with changes in self-reported behavior, but the specific pathways underlying improvement remain uncertain, as the study did not include objective exposure measurements or a control group. Further research using dosimetry and multicenter data is needed to clarify the relationship between objectively measured radiation exposure and dermatologic outcomes.

### Role of Educational Campaigns in Radiation Safety

Educational interventions played a pivotal role in improving radiation awareness and promoting safer practices. The proportion of participants who reported being cautious about radiation exposure increased markedly from 5.8% in 2019 to 70.9% in 2024 (Fig. 2), reflecting the cumulative effect of structured campaigns emphasizing ALARA principles and PPE use. These findings align with prior studies showing that even brief, targeted sessions can significantly enhance protective behaviors among healthcare professionals^8-10^. Brenner and Hall (2007) highlighted the growing need for radiation-safety education in high-exposure specialties such as spine surgery^11^, and ICRP Publication 113 (2009) advocates integrating radiation protection into clinical training^12^. In addition to individual behavior change, practical barriers-such as limited access to well-fitting protective gear and time pressure in emergency procedures-remain challenges in daily practice. Further studies are warranted to explore how these factors may influence self-reported radiation exposure and dermatologic outcomes.

### Experience and Specialization as Risk Factors

Spine surgeons were significantly less likely to show improvement in skin injury (OR, 0.236; 95% CI, 0.069-0.807; p = 0.021), consistent with their higher cumulative exposure during fluoroscopy-guided procedures such as pedicle-screw placement^2,3,13,14^. This study focused on skin injuries, whereas previous studies, including Hijikata et al., evaluated the risk for cataractous changes and chronic inflammatory skin conditions in spine surgeons due to repeated radiation exposure^15^. Radiation-induced skin injury occurred predominantly on the dominant (operating) hand, particularly among right hand–dominant surgeons, supporting earlier findings by Hijikata et al. (2023)^10^. These observations emphasize the need for ergonomic modifications, hand-positioning education, and improved shielding to reduce localized exposure.

### Significance of Radiation-Induced Skin Injury Evaluation and Longitudinal Follow-Up

Ongoing dermatologic screening enables early detection of reversible Grade 1 lesions before progression to irreversible Grade 2 dermatitis or malignancy^4,11,16–19^. The present longitudinal design demonstrated that most mild lesions stabilized or improved, supporting early intervention and continuous monitoring as practical preventive strategies.

### Limitations and Future Directions

This study relied on self-reported weekly fluoroscopy time, which may introduce recall bias.

In addition, self-reported fluoroscopy time may be influenced by the Hawthorne effect, whereby surgeons exposed to repeated educational messaging or survey participation may unintentionally under-report their exposure.

The study was conducted at a single institution, which may limit generalizability due to potential differences in institutional safety culture, availability of protective equipment, and training emphasis. Equipment variability, unmeasured procedural volume, and the pragmatic dermatologic grading system may also affect the findings.

Furthermore, although safety attitudes and self-reported fluoroscopy time improved over the study period, these changes cannot be attributed solely to the institutional educational campaigns. Broader influences—such as generational turnover, national meetings, or prior residency training—may have contributed to improved awareness independent of the intervention.

Despite these limitations, the paired longitudinal design, dermatologist confirmation, and high follow-up rate strengthen internal validity, although objective dosimetry and multicenter validation are needed to fully assess exposure–outcome relationships.

## Conclusion

During the 5-year period in which repeated radiation-safety education was conducted, surgeons demonstrated improved safety attitudes, decreased self-reported fluoroscopy time, and better dermatologist-graded skin findings. These improvements were observed without establishing a causal link, as the study relied on self-reported exposure data and lacked a control group. Further prospective, multicenter studies incorporating objective dosimetry are required to clarify the relationship between actual radiation exposure and dermatologic outcomes and to evaluate the true impact of educational interventions.

## Funding

None.

## Appendix

Supporting material provided by the authors is posted with the online version of this article as a data supplement at jbjs.org (http://links.lww.com/JBJSOA/B82). This content was not copyedited or verified by JBJS.
